# Skin advanced glycation end-products as indicators of the metabolic profile in diabetes mellitus: correlations with glycemic control, liver phenotypes and metabolic biomarkers

**DOI:** 10.1186/s12902-024-01558-9

**Published:** 2024-03-05

**Authors:** Grigorios Christidis, Frederic Küppers, Senem Ceren Karatayli, Ersin Karatayli, Susanne N. Weber, Frank Lammert, Marcin Krawczyk

**Affiliations:** 1 Department of Medicine II, Saarland University Medical Center, Saarland University, Kirrberger Str. 100, 66421 Homburg, Germany; 2 Hannover Medical School (MHH), Hannover, Germany; 3 Laboratory of Metabolic Liver Diseases, Center for Preclinical Research, Department of General, Transplant and Liver Surgery, Medical University of Warsaw, Warsaw, Poland; 4 Endokrinologikum Ulm, Ulm, Germany

**Keywords:** AGE, Diabetes, FGF, GDF15, Liver fibrosis

## Abstract

**Introduction:**

The production of advanced glycation end-products (AGEs) is a key pathomechanism related to the complications of diabetes mellitus. The measurement of HbA1c as one of the AGEs is widely used in the clinic, but also other proteins undergo glycation in the course of diabetes. Here, we measure skin AGEs (SAGEs) in patients with diabetes type 1 (DM1) and type 2 (DM2) and correlate them with metabolic markers as well as non-invasively measured liver fibrosis and steatosis.

**Patients and methods:**

In this cross-sectional study, a total of 64 patients with either DM1 or DM2 and 28 healthy controls were recruited. SAGEs were measured using autofluorescence (AGE Reader). Liver fibrosis and steatosis were quantified using transient elastography, which determines liver stiffness measurement (LSM) and controlled attenuation parameter (CAP). FGF19, FGF21 and GDF-15 were measured in blood samples using ELISA.

**Results:**

SAGEs were elevated in both groups of patients with diabetes as compared to healthy controls (both *p* < 0.001) and were higher in patients with DM2 in comparison to DM1 (*p* = 0.006). SAGEs correlated positively with HbA1c (*r* = 0.404, *p* < 0.001), CAP (*r* = 0.260, *p* = 0.016) and LSM (*r* = 0.356, *p* < 0.001), and negatively with insulin growth factor binding protein 3 (*p* < 0.001). We also detected a positive correlation between GDF15 and SAGEs (*r* = 0.469, *p* < 0.001).

**Conclusions:**

SAGEs are significantly elevated in patients with both DM types 1 and 2 and correlate with metabolic markers, including HbA1c and GDF15. They might also help to detect patients with advanced liver injury in the setting of diabetes.

## Introduction

Diabetes mellitus is a global health concern due to its high prevalence and associated organ complications [1–2]. Whereas hyperglycemia is the hallmark of all diabetes subtypes, the disease progression and complications result from the joint disruption of insulin’s metabolic and hormonal functions, affecting lipid and protein metabolism as well [3–4].

Glycated hemoglobin (HbA1c) is the gold standard for the diagnosis of diabetes and treatment monitoring [5]. However, non-enzymatic glycosylation induced by hyperglycemia can impact a multitude of proteins, lipids, and even nucleic acids [6]. These glycated end-products, collectively referred to as advanced glycation end-products (AGEs), play a pivotal role in the pathogenesis of diabetes, contributing significantly to target organ damage [7]. The post-translational modification of affected proteins involves two successive steps, beginning with the formation of an aldimine (Schiff’s base) via the binding of glucose carbonyl groups to lysine residues’ amino groups [8]; subsequently, this aldimine undergoes an isomerization process known as the Amadori rearrangement, yielding a stable ketoamine [9].

Skin AGEs (SAGEs), assessed non-invasively through ultraviolet-induced autofluorescence, hold promise as indicators of glycemic burden and diabetes-related complications [10]. Research has linked SAGE levels, measured via autofluorescence, to kidney and cardiovascular diseases, vascular complications, retinopathy, mortality, and diabetes risk prediction in susceptible individuals [11–15]. The method has been validated in skin biopsy-based studies in healthy subjects, patients with diabetes and patients with end-stage renal disease [10, 16–17]. However, broader clinical use is hindered by research limitations and knowledge gaps [10], and whether SAGEs and non-enzymatic glycosylation represent unique diabetes features remains unclear. Elevated skin autofluorescence has also been observed in patients with cirrhosis, suggesting AGEs’ potential role in fibrosis progression, possibly mediated through reactive oxygen species (ROS) induction [18–19].

In recent years, two members of the endocrine fibroblast growth factor (FGF) subfamily, namely FGF19 [20] and FGF21 [21], have been shown to be involved in metabolic disorders. FGF19, an enterokine, participates in the regulation of bile acid synthesis and enterohepatic circulation [22]. FGF21, a hepatokine, is involved in carbohydrate and fatty acid metabolism [23] and has been postulated to possess hepatoprotective properties [24]. Additionally, the growth differentiation factor 15 (GDF15) has emerged as a central metabolic biomarker, which is associated with multiple disorders, including cancer [25], cardiovascular diseases [26], and diabetes [27], and has been proposed as a marker for increased mortality risk [28]. Notably, two of these proteins, FGF21 and GDF15, are implicated in energy homeostasis [23] and the pathogenesis of obesity [29], a fact positioning them as potential pharmacological targets [29].

Here we examined SAGE levels in patients with DM1 or DM2, as well as in healthy controls. We aimed to explore potential correlations between SAGE levels and various metabolic markers, non-invasively assessed indicators of liver fibrosis and steatosis, along with serum concentrations of FGF19, FGF21, and GDF15. Specifically, we investigated the connections between SAGEs and liver conditions in the context of diabetes, considering the common occurrence of metabolic dysfunction and liver injury in individuals with diabetes.

## Patients and methods

### Study cohorts

All patients were prospectively recruited from April 2019 to September 2020 in the endocrinological outpatient clinic of the Saarland University Hospital. Overall, 92 subjects were included. Table 1 presents the baseline characteristics of the study cohorts. In total, we recruited 30 patients with DM1, 34 patients with DM2, and 28 healthy subjects as controls. The average duration of diabetes since initial diagnosis was 9.7 years (± 4.4) in the cohort of individuals with DM2 and 24.7 years (± 9.9) in the cohort comprising patients with DM1. All participants were Caucasians and white skinned, without any known dermatological diseases. Blood sampling and laboratory tests were performed in fasted individuals. The study protocol was reviewed and approved by the ethical committee of Ärztekammer Saarland (approval number 84/19). The study was performed in accordance with the Declaration of Helsinki. All recruited subjects provided signed informed consent to participate in the study.

**Table 1:** Basic characteristics of patients with diabetes and controls included in the study

Variable	Controls (*n* = 28)	Patients with diabetes mellitus type 1 (*n* = 30)	Patients with diabetes mellitus type 2 (*n* = 34)
Age (years)	36.8 (+/− 13.2)	47.5 (+/− 14.4)	62.4 (+/− 11.9)
Males (%)	8 (28.5%)	12 (40%)	20 (58.8%)
BMI (kg/m²)	27.7 (+/− 6.4)	27.8 (+/− 5.5)	36.0 (+/− 10.3)
SAGEs	1.58 (+/− 0.43)	2.11 (+/− 0.52)	2.53 (+/− 0.65)
HbA1c (%)	5.33 (+/− 0.35)	8.10 (+/− 1.17)	7.42 (+/− 1.83)
Cholesterol (mg/dl)	181.7 (+/− 45.2)	179.7 (+/− 33.6)	164.4 (+/− 40.1)
LDL (mg/dl)	117.5 (+/− 33.2)	107.9 (+/− 28.8)	95.2 (+/− 36.4)
Triglycerides (mg/dl)	109.2 (+/− 78.3)	106.8 (+/− 79.0)	159.7 (+/− 113.1)
CAP (dB/m)*	251.5 (+/− 69.6)	240.4 (+/− 62.9)	309.4 (+/− 65.1)
LSM (kPa)*	5.9 (+/− 1.9)	4.7 (+/− 1.4)	9.7 (+/− 5.3)
FGF19 (pg/ml)	179.01 (+/− 116.1)	181.7 (+/− 88.7)	123.1 (+/− 86.1)
FGF21(pg/ml)	235.6 (+/− 322.4)	236.7 (+/− 369.7)	390.4 (+/− 394.7)
GDF15 (pg/ml)	441.7 (+/− 335.2)	1005.4 (+/− 996.9)	1840.2 (+/− 1140.8)
IGF1 (ng/ml)	146.4 (+/− 48.4)	111.6 (+/− 39.9)	120.65 (+/− 43.5)
IGFBP3 (µg/ml)	5.48 (+/− 0.9)	4.1 (+/− 1.2)	4.08 (+/− 1.3)

All results are presented as mean (+/−SD), unless stated otherwise

Abbreviations: BMI: body mass index, CAP: controlled attenuation parameter, dB/m: decibel/meter, FGF19: fibroblast growth factor 19, FGF21: fibroblast growth factor 21, GDF15: growth differentiation factor 15, HbA1c: glycated hemoglobin cluster 1c, IGF1: insulin like growth factor 1, IGFBP3: insulin like growth factor binding protein 3, kPa: kilopascal, LDL: low density lipoprotein, LSM: liver stiffness measurement SAGEs: skin advanced glycation endproducts

*LSM and CAP available for 85 individuals

### Measurements of skin AGEs (SAGEs)

The measurement of SAGEs was performed using the AGE Reader (Diagnoptics, Groningen, Netherlands). The AGE Reader allows the non-invasive measurement of SAGEs based on their divergent fluorescence characteristics [30]. Autofluorescence is the natural phenomenon of emission of light by a structure, after an initial phase of light absorption. The values of the measured AGEs were adjusted with reference values defined for the respective age groups of the patients provided by the manufacturer. In brief, the measurements are performed on dry and clean skin of the patient’s forearm without optical contamination such as tattoos. The measurement lasts approximately 12 s.

### Quantification of liver fibrosis and steatosis by liver stiffness measurements (LSM)

Liver stiffness measurement (LSM) was performed using FibroScan® (EchoSens, Paris, France). Transient elastography (TE) provides liver stiffness measurement of liver parenchyma based on the physical properties of fibrotic tissues visualized by conventional ultrasound technique. Together with LSM, quantification of liver steatosis using controlled attenuation parameter (CAP) was performed. LSM and CAP median values were obtained based on at least 10 valid measurements. All measurements were considered as valid when the IQR/median ratio was below 0.3.

### Bioelectrical impedance analysis (BIA)

Bioelectrical impedance analysis (BIA) was performed with the medical body composition analyzer (mBCA 515, Seca, Hamburg, Germany). The fundamental of the bioimpedance analysis is that different biological tissues have different electrical properties. Thus, the distinct impedance of a biological structure, meaning the impediment to the flow of an electrical current, can provide information for the tissues and fluids, which it consists of [31].

### Measurements of serum FGF19, FGF21 and GDF15

The measurements of FGF19, FGF21 and GDF15 in sera of fasted patients were performed using human FGF19, human FGF21 and human GDF15 Quantikine ELISA kits by R&D Systems (Minneapolis, Minnesota, USA), according to the manufacturer’s instructions.

### Measurements of hormone concentrations

Fasting venous serum and plasma samples from all patients were collected between 08:00–10:00 am in order to avoid circadian fluctuations, followed by hormone measurements of cortisol, adrenocorticotropic hormone (ACTH), thyroid stimulating hormone (TSH), free triiodothyronine (fT3) and tetraiodothyronine (fT4), insulin like growth factor 1 (IGF1), and insulin growth factor binding protein 3 (IGFBP3). For all subjects, glycated hemoglobin A1c (HbA1c), glucose, cholesterol, triglyceride, low density lipoprotein (LDL) and high-density Lipoprotein (HDL) blood levels were also determined.

### Statistical analysis

Statistical analyses were performed using IBM SPSS version 28.0 (IBM, Armonk, New York, USA). The distribution of data was checked using the Kolmogorov-Smirnov test. One-way ANOVA test was used for normally distributed variables, and the Kruskal–Wallis test was applied to parameters with non-parametric distribution. A post hoc-test with Bonferroni correction was performed, if significant differences between the groups were found. Two normally distributed variables were compared with Student’s *t*-test. Pearson correlation coefficient was used to detect bivariate correlations. Data were reported as mean and SD or median and range. P-values < 0.05 were considered to be statistically significant.

## Results

### Levels of SAGEs in patients with diabetes mellitus

**Fig. 1:**
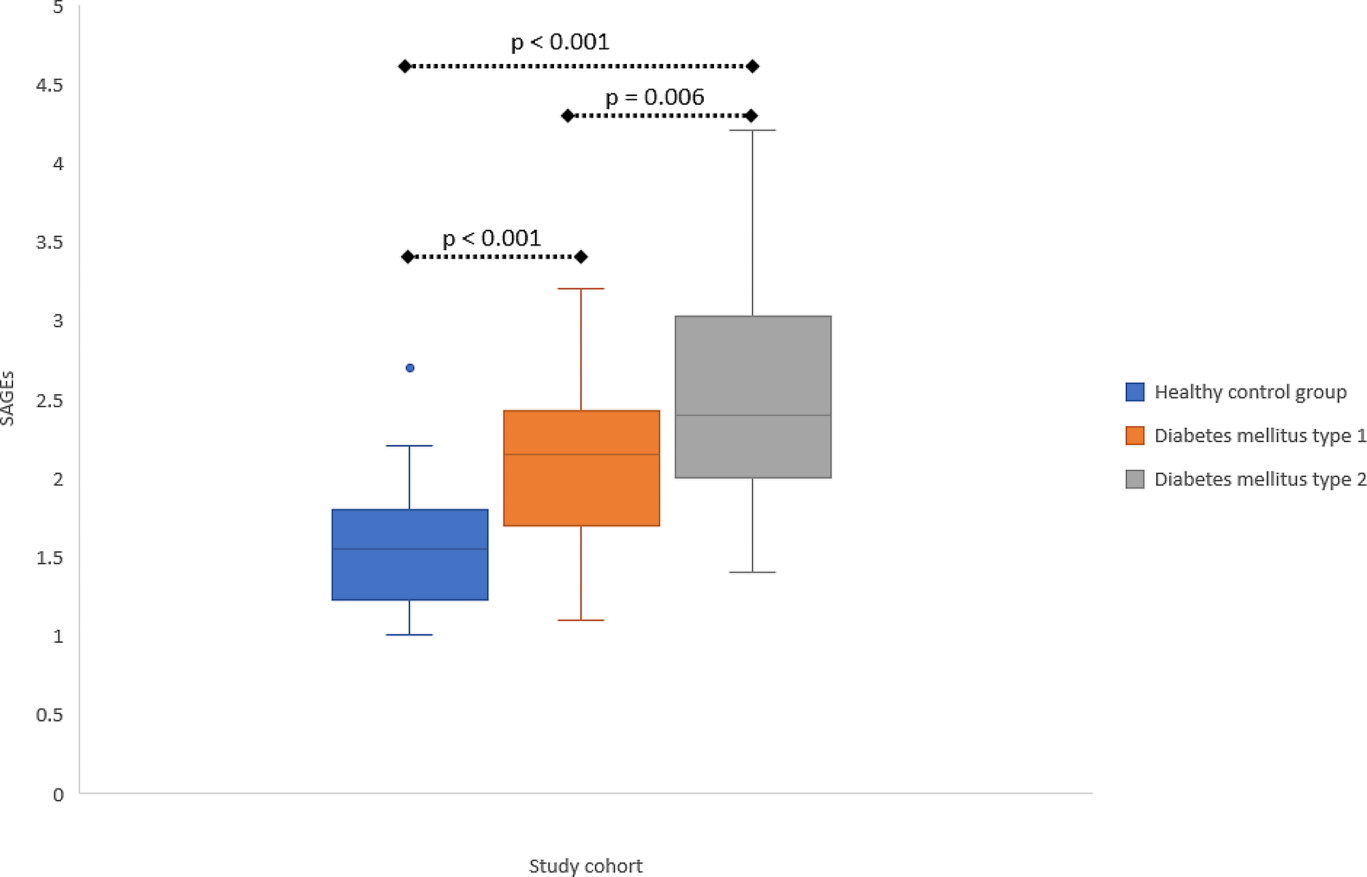
Skin AGEs in patients with diabetes and healthy subjects

**Fig. 2:**
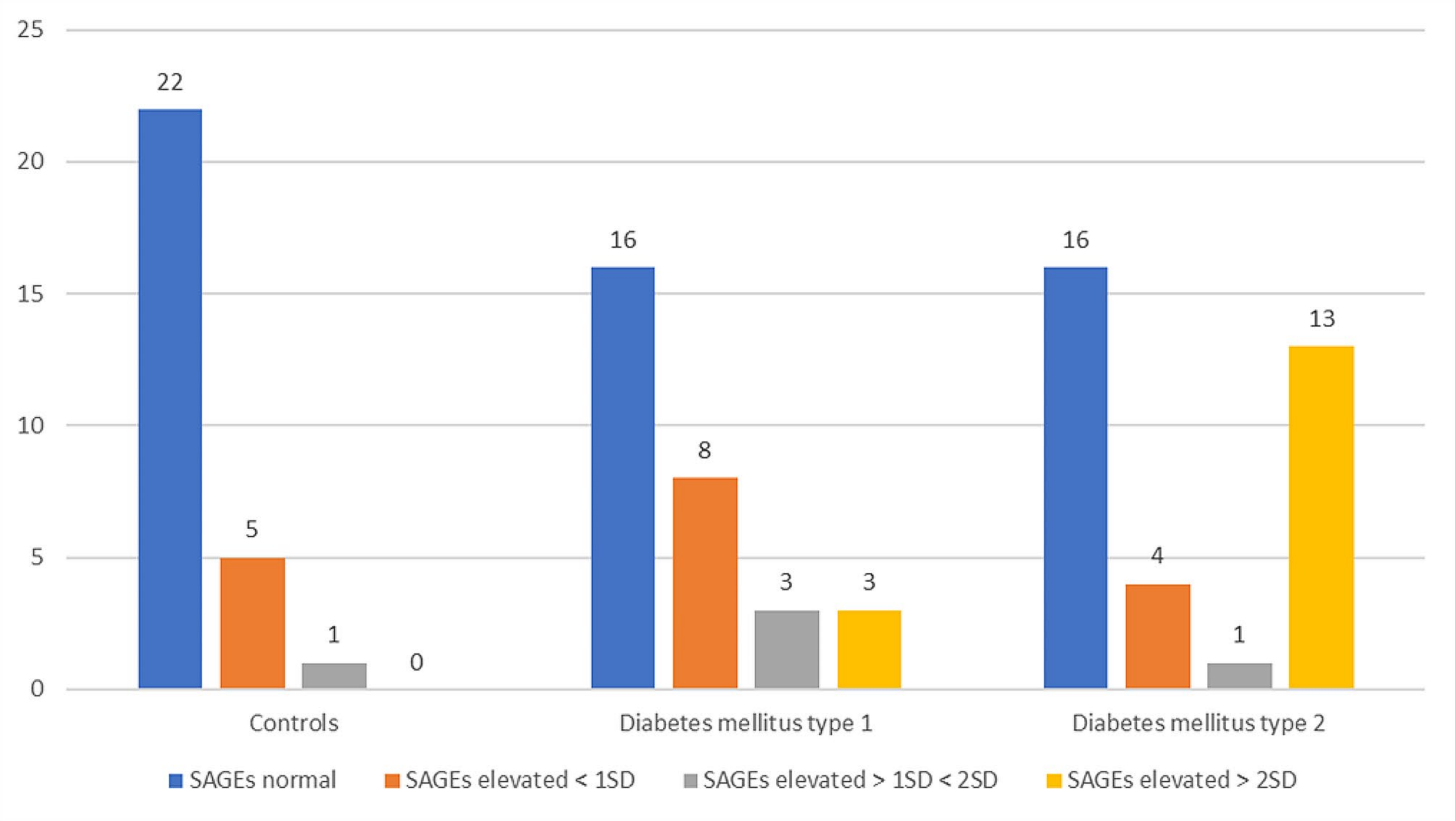
Number of individuals with normal and elevated age-adjusted SAGE values among healthy subjects, patients with diabetes mellitus type 1 and with diabetes mellitus type 2

**Fig. 3:**
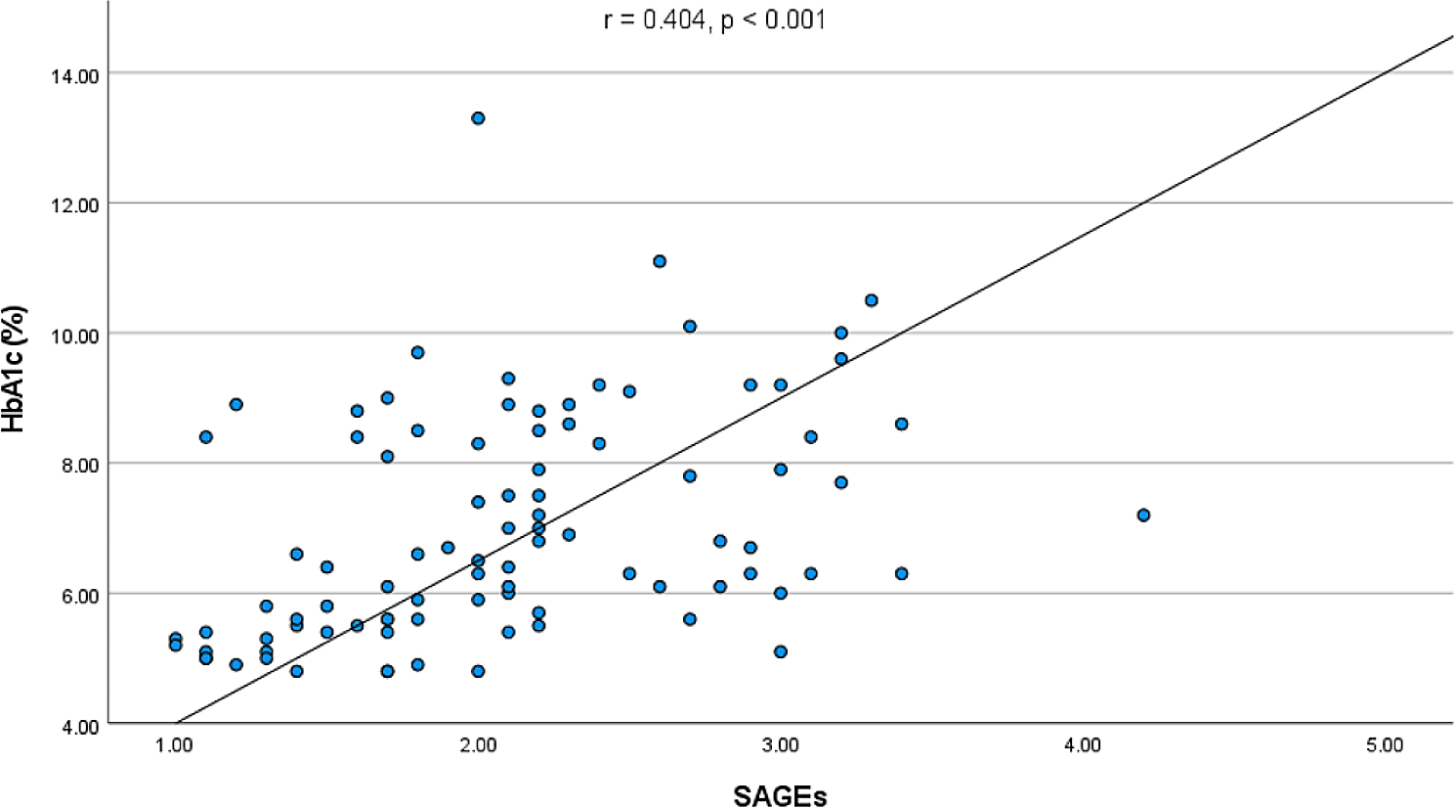
Correlation between skin AGEs and HbA1c in patients with diabetes type 1 and 2

Significantly higher SAGEs were detected in both groups of patients with DM as compared to healthy controls (both *p* < 0.001; Fig. 1). As illustrated in Fig. 1, SAGEs were significantly (*p* = 0.006) higher in patients with DM2 as compared to those with DM1. This difference could mirror the younger age of the latter group (Table 1). Since the reference values of AGEs could depend on the age of the examined person, we examined the proportion of the subjects in each group with SAGE values within the age-adjusted reference values. This fraction was 78.6% in the control group, 53.3% in the group with DM type 1 and 47.1% in the group with DM type 2, consistent with a significant (*p* = 0.001) difference between the groups (Fig. 2). Moreover, we detected a positive correlation of measured SAGEs with HbA1c, independently of the diabetes type (*r* = 0.404, *p* < 0.001; Fig. 3).

### SAGEs correlate with liver stiffness in patients with diabetes mellitus type 2

Patients with DM2 had the highest BMI among the three study groups (*p* < 0.01). The muscle mass was lower in the DM2 group in comparison with the two other groups, although not statistically significantly between DM1 and DM2 (DM2 vs. control group *p* = 0.026; DM2 vs. DM1 *p* = 0.132). Patients with DM2 presented with most advanced liver steatosis and fibrosis, as reflected by the highest CAP and LSM values, respectively. Of note, we did not detect any difference between patients with DM type 1 and controls in terms of BMI, CAP, or LSM.

**Fig. 4:**
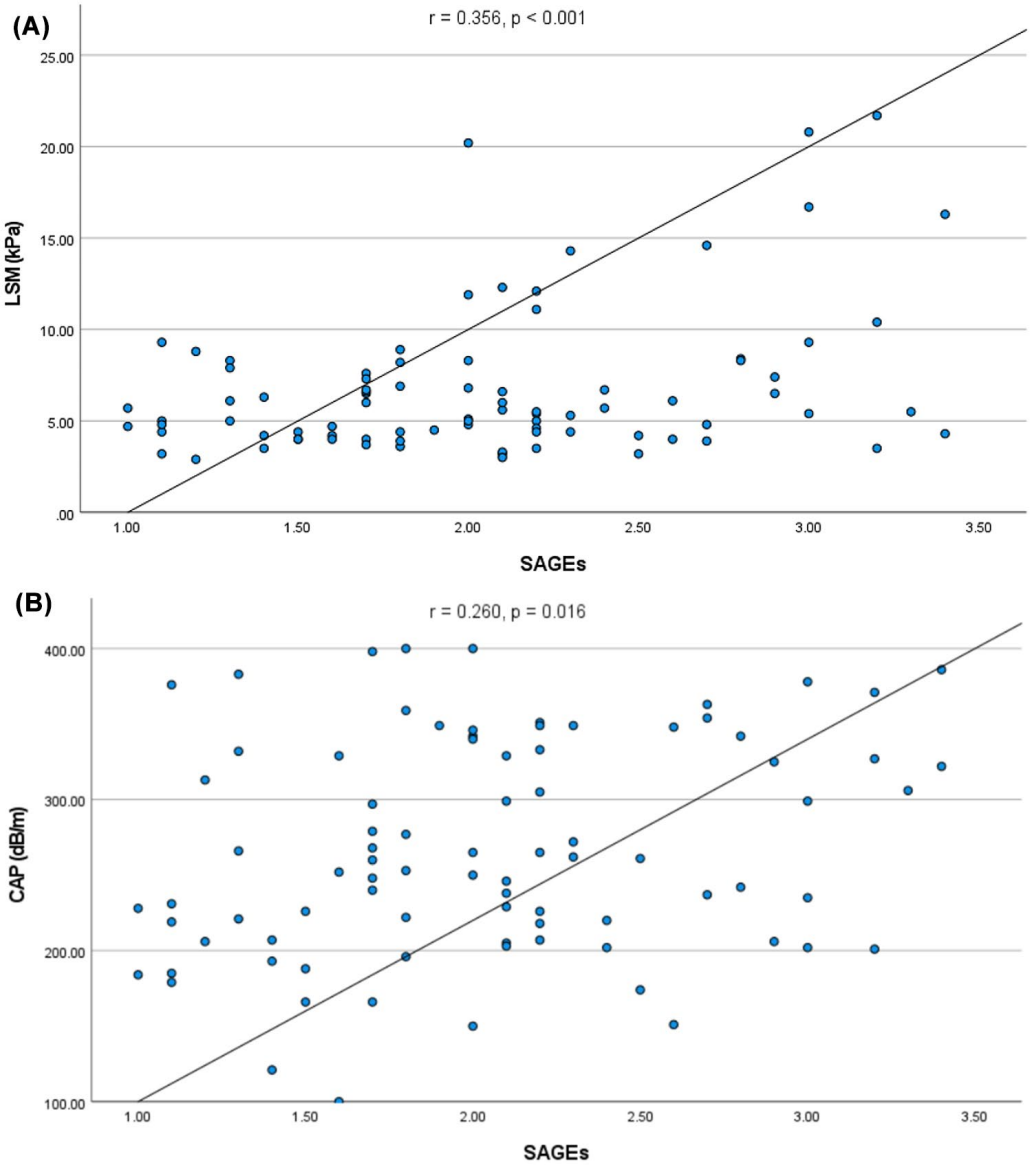
Correlation between skin AGEs, liver stiffness measurement (LSM) (**A**) and controlled attenuation parameter (CAP) (**B**)

As presented in Fig. 4, SAGEs correlated with both LSM (*r* = 0.356, *p* < 0.001), and CAP values (*r* = 0.260, *p* = 0.016) in the entire study cohort. In total, 39% of recruited individuals showed CAP values greater than 288 dB/m, which is suggestive of fatty liver [32]. We detected a trend (*p* = 0.05) towards higher SAGE levels in this group as compared to individuals with CAP values below the cut-off.

**Fig. 5:**
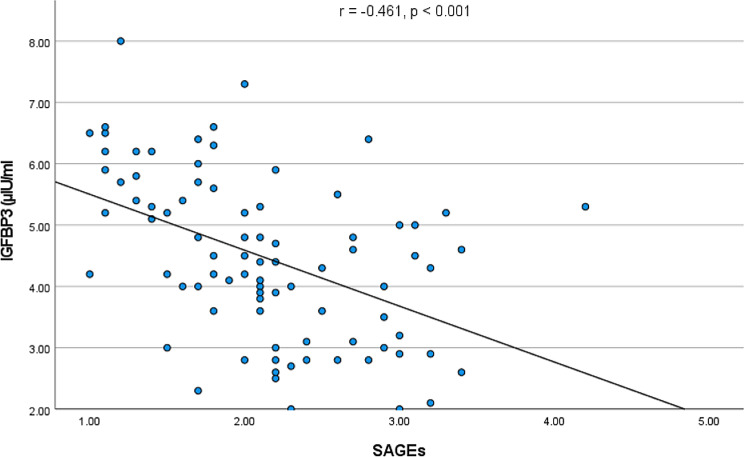
Negative correlation between skin AGEs and insulin growth factor binding protein 3 (IGFBP3)

We detected significant correlations of SAGEs with BMI (*r* = 0.299, *p* = 0.04) and fat mass (*r* = 0.259, *p* = 0.014), but not with muscle mass (*p* = 0.59). Of note, SAGEs correlated negatively (*p* < 0.001) with IGFBP3 serum concentrations. (Fig. 5). IGFBP3 levels were significantly (*r* = − 0.461, *p* < 0.001) decreased in both groups of patients with DM, as compared to the control group. There was no significant correlations between SAGEs and lipid levels, ACTH, cortisol, TSH, fT3 and fT4 or activities of ALT and AST.

### FGF19, FGF21 and GDF15 levels in patients with different types of diabetes and their correlation with SAGEs

**Fig. 6:**
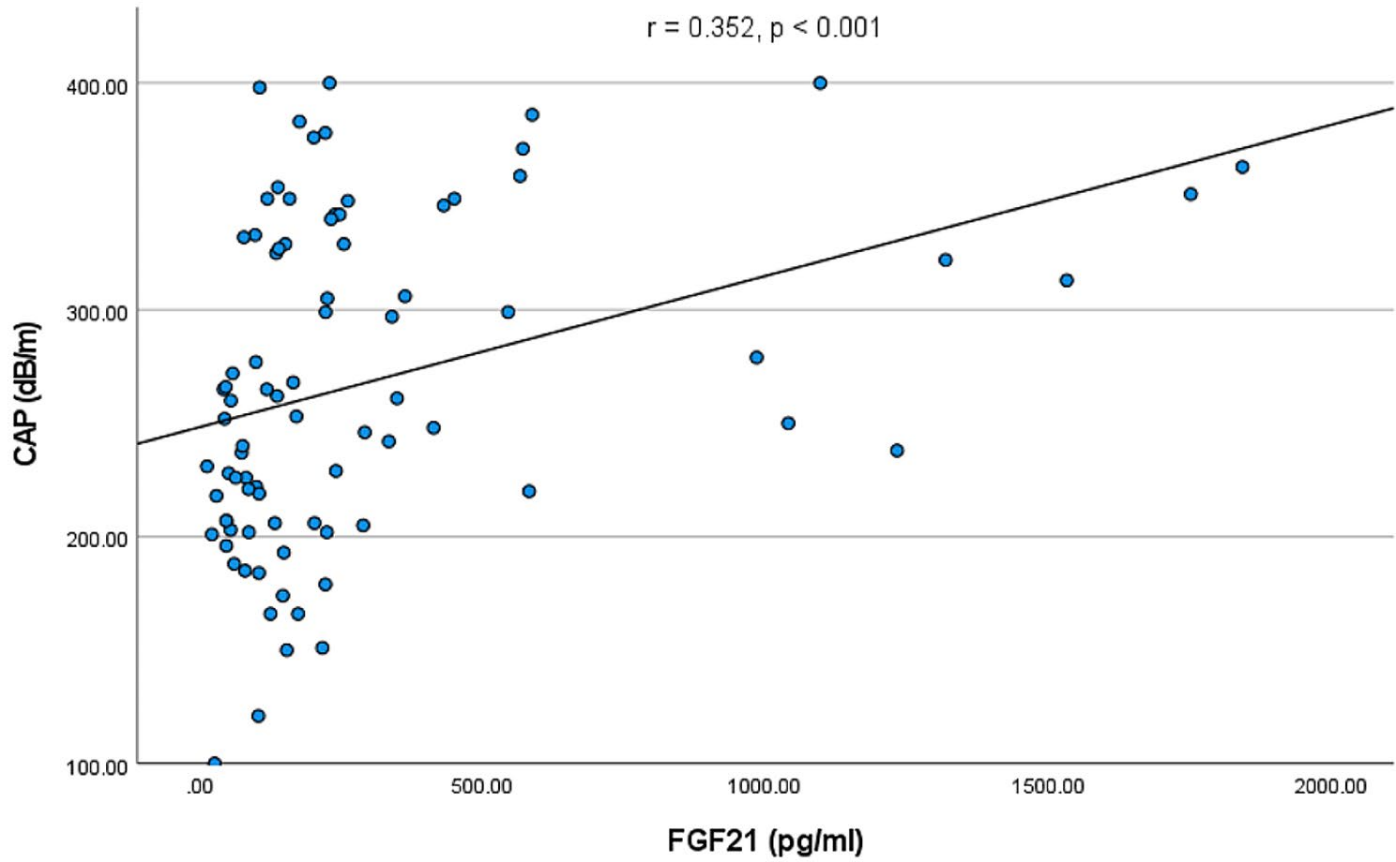
Correlation between FGF21 and liver steatosis measured by controlled attention parameter (CAP)

We observed a significant elevation of GDF 15 in the group of patients with DM1 and DM2, as compared with healthy controls (*p* = 0.007 and *p* < 0.001, respectively), and GDF15 levels correlated significantly with SAGEs (*r* = 0.469, *p* < 0.001). FGF19 serum levels were significantly (*p* = 0.012) lower in patients with DM type 2 as compared with DM type 1. In the case of FGF21, we observed a significant elevation in the DM type 2 group, as compared with DM type 1 and the healthy control group (*p* = 0.003 and *p* = 0.012, respectively). Furthermore, we ascertained a positive correlation between the serum levels of FGF21 and CAP values (*r* = 0.352, *p* < 0.001) (Fig. 6), indicating that fat accumulation in the liver induces the production of this hepatokine. On the other hand, we did neither detect any significant correlations between SAGEs and FGF19 nor FGF21 (all *p* > 0.05).

## Discussion

In the current study, we combined non-invasive quantification of advanced glycation end-products in skin with measurements of liver stiffness and steatosis as well as select serum hepatokines in patients with diabetes. First of all, we showed that SAGEs correlate with liver stiffness both in patients with diabetes and in normoglycemic individuals. Our data replicate the previously suggested correlation between SAGEs and HbA1c [33]. More crucially, we were also able to find consistent correlations between the increased levels of GDF15 and the measured skin AGEs.

Although liver biopsy is commonly regarded as the gold-standard method to quantify liver fibrosis, transient elastography has gained its place as a valid, non-invasive method that allows measurements of liver scarring and steatosis. Our study reproduces the correlation between liver stiffness and AGEs from the previous studies [34–38] but extends the correlation to skin AGEs specifically to individuals with diabetes type 1 and 2. This correlation seems to be independent of HbA1c, given that a significant correlation between HbA1c and LSM was not observed. Of note, SAGEs did not correlate with the non-invasively quantified hepatic fat in our patients with fatty liver disease, although a trend was detected. Of note, there was a significant correlation of SAGEs with CAP values, when all measurements were included and the entire cohort was analysed. This finding is consistent with the previously published data in patients with fatty liver from Brazil where hepatic steatosis was quantified using abdominal ultrasound [34]. The authors of that study demonstrated a continuous elevation of AGEs in serum autofluorescence from the early stage of fat accumulation in the liver [34]. We reckon that larger cohorts of patients with CAP-based measurements of liver fat contents might be required to further investigate the association between hepatic steatosis and SAGEs. We nevertheless detected a significant correlation with BMI and fat mass in the bioimpedance analysis, which further indicates that obesity or excess fat tissues could significantly influence the process of advanced glycation in the skin.

We analysed correlations between skin AGEs and the endocrine fibroblast growth factors with pivotal metabolic role, FGF19 [22] and FGF21 [23] and GDF 15, which is also considered to be a biomarker induced mainly under metabolic distress, stress conditions or inflammatory processes [39]. We found a progressive elevation of GDF15 between the three groups: the control group had the lowest blood levels of GDF15, patients with diabetes mellitus type 1 significantly elevated levels as compared to the control group and patients with diabetes type 2 showed the highest serum GDF15 as compared to both groups. These findings are in accordance with previous published data which show increases in circulating GDF15 in patients with DM2 [40] and that even the effect of metformin in this group of patients could be at least partly induced via GDF15 signalling [41]. Serum GDF15 correlated with the skin AGEs. Collectively, these findings enhance the notion, that the detection of increased SAGEs indicates established advanced metabolic alterations, especially in the context of diabetes mellitus type 2 and metabolic syndrome. We did not detect a significant association between SAGEs and FGF21. However, the elevation of circulating FGF21 in patients with diabetes mellitus type 2 compared with the two other groups is consistent with previous data [23]. However, it remains unclear whether this elevation is due to the beneficial metabolic role of FGF21 in the setting of diabetes and obesity or represents a resistance state in this context. Overall, we can assume that skin autofluorescence not only successfully detects the presence of diabetes mellitus and its glycaemic and metabolic alterations but is also associated with circulating biomarkers, in our study GDF15, the circulating levels of which have been associated in previous studies with the severity of diabetes and the presence of potential diabetic complications [42–43].

The last finding of unclear significance is the negative association of SAGEs with IGFBP3. In our study, serum IGFBP3 was significantly reduced in both cohorts of diabetic patients. To the best of our knowledge, there are no previous findings in the literature which associate the IGFBP3 with elevated AGEs in the skin autofluorescence. There are though some data indicating that increased levels of IGFBP3 are associated with diabetic cardiomyopathy and that suppression of IGFBP3 is associated with improved cardiovascular health [44]. More interesting is the fact that elevated levels of IGFBP 3 are consistently associated with some types of cancers, especially breast, ovarian, colorectal, lung and brain cancers [45–50]. Our observations concerning IGFBP3 are rather unexpected and mandate further investigation.

Our study has certain limitations. First of all, the size of our cohorts is relatively small. Secondly there are many different groups of SAGEs, some of them nonfluorescent, which cannot be quantified or differentiated from the existing devices. We conducted the study in white-skinned Caucasians with non-previously diagnosed skin disease and tattoos. So, it remains unclear, if race and skin pathologies could influence or alter the above-mentioned results. It is impossible to adjust our result to the current medication of the patients, especially those with diabetes type 2. It is consequently difficult to estimate potential direct or indirect influences of medicines intake on SAGEs. We analyzed associations between SAGEs and liver fibrosis and steatosis based on non-invasive transient elastography. Although liver biopsy-based histopathology is regarded as the gold-standard method of determining status of hepatic health and disease, we do not have histopathology data on any of the patients in this study. We also do not have full data on biochemical indicators of liver fibrosis (e.g., FIB-4 or ELF) in recruited patients or controls. Finally, our primary research emphasis was directed towards skin AGEs. Consequently, we did not concurrently assess serum AGE levels, thereby precluding us from drawing definitive correlations between these two variables.

Overall, our study provides compelling evidence that SAGEs correlate with stages of liver fibrosis, quantified via transient elastography, serum HbA1c and GDF15, which is a biomarker of advanced metabolic derangements. Measurement of skin AGEs via skin autofluorescence is a feasible, practical, reliable and cost-effective method to assess the presence of diabetes mellitus and associated metabolic complications and liver pathology. We reckon that SAGEs might be used in the future to detect patients with diabetes in the general population and patients with liver injury in the diabetic population. In this context, it would be interesting to further investigate potential associations between SAGEs and insulin resistance, even in the absence of manifest diabetes mellitus. Measurements of the skin autofluorescence in the routine clinical praxis could add further valuable information to the clinical and biochemical assessment of patients with diabetes mellitus and predict the risk of cardiovascular, metabolic, inflammatory and even malignant diseases. These preliminary observations will however require replications in larger groups of normoglycemic and diabetic individuals.

## Data Availability

The datasets used or analysed during the current study are available from the corresponding author on reasonable request.

## Abbreviations

ACTH: adrenocorticotropic hormone
AGE: advanced glycation endproduct
BIA: bioelectrical impedance analysis
CAP: controlled attenuation parameter
DM: diabetes mellitus
FGF: fibroblast growth factor
GDF: growth differentiation factor
HbA1c: glycated hemoglobin A1c
HDL: high density lipoprotein
IGF: insulin-like growth factor
IGFBP: insulin-like growth factor binding protein
IQR: interquartile range
LDL: low density lipoprotein
LSM: liver stiffness measurement
SAGE: skin advanced glycation endproduct
TE: transient elastography
TSH: thyroid stimulating hormone
